# High-Resolution Computed Tomography Scores in Cases of Bronchopulmonary Dysplasia

**DOI:** 10.1155/2022/5208993

**Published:** 2022-02-07

**Authors:** Abdullah Barıs Akcan, Seyhan Erişir Oygucu, Ahmet Gökhan Arslan, Deniz Özel, Nihal Oygür

**Affiliations:** ^1^Department of Pediatrics, Division of Neonatology, School of Medicine, Aydın Adnan Menderes Universıty, Turkey; ^2^Department of Pediatrics, University of Kyrenia, Cyprus; ^3^Department of Radiology, School of Medicine, Akdeniz University, Turkey; ^4^Department of Biostatistics and Medical Informatics, School of Medicine, Akdeniz University, Turkey; ^5^Department of Pediatrics, Division of Neonatology, School of Medicine, Akdeniz University, Turkey

## Abstract

**Background:**

Bronchopulmonary dysplasia (BPD) carries a risk of long-term pulmonary sequelae. High-resolution computed tomography (HRCT) is a method of detecting such structural changes. This study is aimed at characterizing structural abnormalities associated with BPD and at evaluating the clinical findings in the newborn period associated with HRCT scores.

**Methods:**

28 patients born with a mean gestation age of 30 ± 2.9 weeks and diagnosed as BPD in their neonatal period were reevaluated when they were between the postnatal ages of 6 and 12 months. HRCT was performed in 20 patients with a history of moderate and severe BPD. Scans were interpreted by one radiologist using a scoring system.

**Results:**

Patients were 9.8 ± 2.3 months at the time of reevaluation. The average HRCT score of patients was, respectively, 7.20 ± 4.05 with moderate and 7.40 ± 2.84 with severe BPD. The difference between them was not significant (*p* = 0.620). When moderate and severe groups were collected as a whole on the basis of physical findings and drug treatment, 6 had normal physical examination findings, no oxygen and no drug requirement; 14 had at least one finding at the time of reevaluation. No significant difference was detected in terms of HRCT score between the two groups (6.50 ± 3.83 versus 7.64 ± 3.30).

**Conclusions:**

More studies are needed in terms of the role of HRCT in the assessment of BPD prognosis. A contemporary definition of BPD that correlates with respiratory morbidity in childhood is needed. Also, a new lung ultrasound technique for predicting the respiratory outcome in patients with BPD can be used instead of HRCT.

## 1. Introduction

Northway et al. [1] made the definition of classical bronchopulmonary dysplasia (BPD) for the first time in 1967. This definition, now considered as the old BPD, described a common small airway disease characterized by severe damage to large airways, interstitial and alveolar oedema, fibrosis, and hyperventilation areas that developed in newborns born between 30 and 37 gestation weeks [1]. In this type of description, BPD is mostly linked to oxygen toxicity and barotrauma, which is the result of preterm delivery babies ventilated with high pressure and oxygen due to respiratory distress syndrome (RDS). However, in recent years, better treatment of antenatal steroid, the use of prophylactic surfactant treatment, and modern ventilation techniques have begun to offer survival to preterm infants with very small gestational age and a new concept of BPD has emerged in these groups of preterm infants. With this definition, referred as the “new BPD,” their RDS are initially absent or mild. Small preterm infants with new BPD have shortage in gas exchange areas, low capillary vessels and alveolar numbers, less emphysema, and minimal fibrosis, but more diffuse damages are seen in the lung parenchyma [2, 3].

BPD also causes significant changes in the pulmonary mechanics, with an average of 50% of cases taken to the hospital due to lower respiratory tract infection, within the first year after discharge [4]. Follow-up data on structural changes associated with new BPD are still sparse. High-resolution computed tomography (HRCT) is a much more sensitive method of detecting such structural changes. HRCT scans at infancy after new BPD reveal abnormalities [5, 6]. Recent evidence suggests that the lungs grow partly by neoalveolarization throughout childhood and adolescence. This has important effects since developing lungs have the potential to recover from early life insults and respond to emerging alveolar therapies [7].

Our aim was to characterize structural abnormalities associated with BPD and to evaluate the clinical findings in the newborn period associated with HRCT scores.

## 2. Methods

### 2.1. Subjects and Data Collection

Twenty-eight cases who were diagnosed as BPD in the neonatal period were enrolled from the Akdeniz University Faculty of Medicine (AUFM), Neonatology Department, and the private and public hospitals in Antalya. The evaluation was made when they were between the postnatal ages of 6 and 12 months. Demographic and clinical data about their medical history was obtained from their medical charts. Patients were divided into three groups according to clinical and radiological characteristics in their newborn period as mild BPD (eight subjects), moderate BPD (ten subjects), and severe BPD (ten subjects) according to the definition of BPD diagnostic criteria [2]. Cases with BPD who were diagnosed with congenital heart disease and cases who were not adequately matched during echocardiographic examinations were excluded from the study. In addition, considering the ethical reasons, lung scans (HRCT) were not performed for cases with mild clinical scoring. Echocardiographic examination was performed in all cases for the evaluation of the cardiovascular system. For the evaluation of the respiratory system, HRCT was recorded in cases with moderate and severe clinical scoring.

### 2.2. Ethical Statement

This study was conducted in accordance with the Declaration of Helsinki and was approved by the Akdeniz University Medical Faculty Ethical Board (Decision no. 5186). Written informed consent was obtained from the parent or legal guardian of each participating case.

### 2.3. HRCT Analyses

20 cases, who agreed to participate in the HRCT study, were examined with Toshiba X-vision spiral computed tomography, by a single radiology specialist at the Department of Radiology. The expert who made the evaluation had no knowledge about the BPD classification of the cases.

In the HRCT protocol, the scan area from the lung apex to the end of the lung parenchyma was scanned with a 1 mm cross-sectional thickness and a 7 mm cross-sectional area. The cross-sectioning time was 0.4 mm, 120 kV, 150 mA (60 mAs) in the scan, and high geometric resolution algorithm (FC50) was used in the reconstruction. From the cases, it was not asked to hold their breath because they were children; the intended examination was possible when they were kept as stationary as possible. Images were printed to the parenchymal window with -700 Hounsfield Unit (HU) window level (WL) and 1400 HU window width (WW) and the mediastinal window with 40 HU WL and 400 HU WW. An experienced radiologist did patients' HRCT reporting both on film and at workstations (Vitrea®). HRCT was evaluated by the new HRCT scoring system that was developed by Ochiai et al. [8]. According to this scoring system, HRCT findings were evaluated in three categories (Table 1).

#### 2.3.1. Hyperexpansion

Hyperexpansion is accepted as the excessive expansion of the lung into the intercostal bulging or the intercostal space. In this category, the mosaic pattern formation, namely, the visible aeration difference in lung areas, is evaluated [8].

#### 2.3.2. Emphysema

The areas of emphasis separated by sharp boundaries are defined as bulla. Bullae are classified by number and size, and their sizes are evaluated according to their diameters > 5 mm or ≤5 mm [8].

#### 2.3.3. Fibrosis

Central abnormalities are assessed as malformations and thickening of the bronchovascular structure. Peripheral abnormalities have been accepted as triangular subpleural opacities, and consolidation is defined as an increase in attenuation that erases the underlying vessels. Scoring is done according to the severity of the malformations and thickening of the bronchovascular structure, according to the number of lobes in the form of triangular-shaped subpleural opacities and whether or not the consolidation is significant [8].

#### 2.3.4. Subjective Evaluation

The radiologist has scored the cases as mild, moderate, and severe according to their own personal assessments, without clinical knowledge.

### 2.4. Statistical Analyses

In the study, statistical analyses of the subjects and control group data were performed using the “Statistical Package for Social Sciences (SPSS for Windows 15.0) (Chicago, USA)” program. The McNemar test and the kappa compliance statistics were used to compare radiological scoring of the cases in the 6-12^th^ months whose BPD classifications were moderate and severe in the newborn period. In the study, parametric distributed variables were assessed by the Student *t*-test and nonparametric variables by the Mann–Whitney *U* test. Categorical variables were assessed by the chi-square test or Fisher's exact test. Statistical significance was accepted as *p* < 0.05. There is powerful correlation if Spearman's correlation coefficient (*r*) is higher than 0.50 and weak correlation if it is between 0.25 and 0.50; if it is less than 0.25, there is no correlation; if it is negative, it is considered as an inverse correlation.

## 3. Results

### 3.1. Neonatal Period Results

13 (46.4%) of the cases were male and 15 (53.6%) were female. 15 (53.6%) were proceeded by AUFM, and 13 (46.4%) were proceeded by other centers. The gestational ages were 30 ± 2.9 weeks according to the data of the files, and 4 (14.28%) were younger than 28 weeks. Birth weights were 1372 ± 784 grams, and 12 (42.8%) were below 1000 grams. The duration in the mechanical ventilation was 22.9 ± 16 days, the duration in the CPAP (continuous positive airway pressure) was 15.3 ± 4.2 days, and the time of the oxygen with hood was 12.0 ± 4.2 days. The duration of total oxygen support was calculated as 50 ± 16.7 days. Clinical BPD staging was performed on 28 cases. Eight of them (28.6%) were mild, ten (35.7%) were moderate, and ten (35.7%) were severe BPD. Cases with moderate and severe BPD stayed 26.6 ± 17.3 days in the mechanical ventilation, 16.7 ± 3.5 days in CPAP, and 11.7 ± 3.9 days in the hood oxygen support. The duration of total oxygen support was calculated as 54.9 ± 17.4 days (Table 2).

### 3.2. Results between the 6^th^ and 12^th^ Months

The ages of the cases were 9.8 ± 2.3 months. HRCT was performed on 20 of the 28 cases with moderate BPD (*n*: 10) and severe BPD (*n*: 10) according to the clinical BPD stage [2]. The HRCT scoring results of the cases are given in Table 3. The average HRCT total score of the cases with moderate BPD was 7.20 ± 4.05 and that of the cases with severe BPD was 7.40 ± 2.84. The difference between the HRCT scores of the moderate and severe BPD groups was not significant (*p* = 0.620).

The relation between some respiratory system data of the cases in the neonatal period and between 6 months and 1 year and the HRCT total score values was investigated. According to the BPD scoring performed during the neonatal period, there was no significant correlation between the duration of oxygenation, the duration of mechanical ventilation (MV), birth weights, and the total HRCT scores for cases with moderate and severe BPD (respectively, *p* = 0.408 and *r* = −0.196 for the duration of oxygenation, *p* = 0.836 and *r* = −0.05 for the mechanical ventilation duration, and *p* = 0.439 and *r* = 0.183 for birth weights). It was determined that diuretics were given to all the cases. There was also no significant difference in HRCT total scores between those who applied steroids and those who did not (*p* = 0.789) (Table 4).

There was no significant difference in HRCT total scores between those who had normal and pathologic respiratory findings and those who use respiratory system medication and those who do not use any medication (*p* = 0.592 in terms of respiratory findings, *p* = 0.497 in terms of drug use).

The average HRCT total score of 20 cases with moderate and severe BPD and 6 cases who had normal physical examination findings when they were called for the control between 6 months and 1 year, who had no oxygen requirement and who had no drug use, was 6.50 ± 3.83. The average HRCT total score of 14 cases with at least one finding was 7.64 ± 3.30. There was no significant difference between the two groups in terms of HRCT total score (*p* = 0.43).

**Figure 1:**
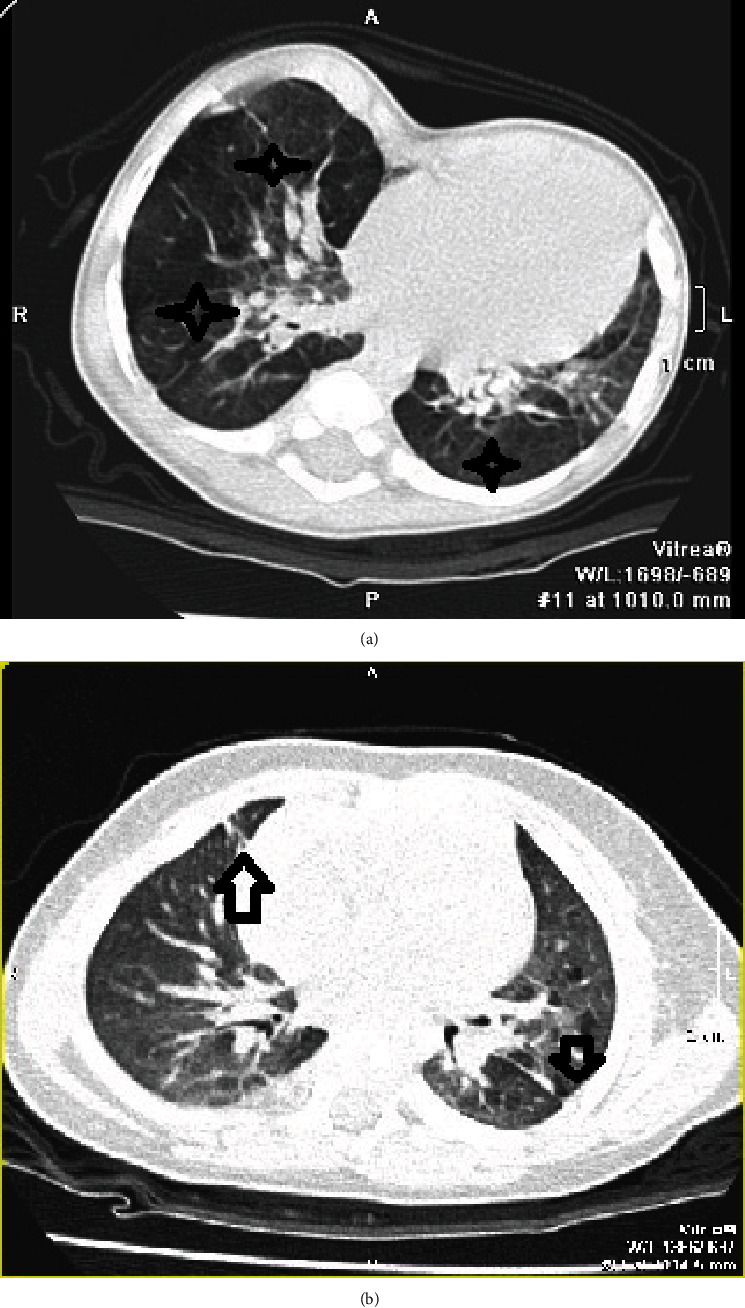
The most common abnormalities in HRCT scans. (a) Stars: the decreased pulmonary attenuation (mosaic perfusion). (b) Arrows: linear/triangular subpleural opacity.

The most common abnormalities in HRCT scans appear in Figure 1.

## 4. Discussion

In recent years, scientific and technical developments in neonatology have led to a survival rate of <10% to >80% for low birth weight premature infants in developed countries; however, this development has led to the appearance of more morbidities [9–11]. The pathogenesis of BPD is multifactorial, oxygen toxicity, barotrauma, volutrauma, and even biotrauma, where IL-1*β*, IL-6, and IL-8 play a part in the aetiology [12, 13]. It is accepted that the frequency increases as the gestational age and the birth weight decrease and the risk, especially at preterms born less than 30 weeks of gestation, is reported to be higher [14, 15]. The average duration of total oxygen support of our cases was calculated as 50 ± 16.7 days; it has been determined that there are a lot of oxygen supply periods exceeding the classical identification criterion for the oxygenation time of 28 days and more too much. In preterm infants with moderate and severe BPD, the duration of mechanical ventilation or the duration of CPAP was found longer and there is an idea that the prolongation of respiratory support will increase the barotrauma and will aggravate the BPD or that severe BPD will require longer ventilation [16–20]. Long-term cardiac and respiratory prognosis of newborns with BPD is still unknown. It has been emphasized that screening of the lungs by computed tomography in the studies is useful for BPD diagnosis and especially opting for HRCT in evaluating pulmonary parenchyma will be useful in differential diagnosis [21–23]. HRCT gives a more detailed picture of the airway size and wall thickness, and with this method, information about hyperinflation due to air entrapment, oedema, and fibrosis can be obtained. HRCT is considered as a much more sensitive examination for detecting structural changes in the lung [2, 21–26]. Radiological findings in the evaluation of BPD with HRCT should be done with a certain scoring and that seems important to compare the data. For this purpose, researchers developed different HRCT scorings for BPD and usually used parameters such as linear, subpleural, triangular opacities, mosaic pattern, and hyperexpansion as scoring parameters [8, 27, 28]. In most of the studies related to HRCT evaluations in the older ages of cases with BPD in the neonatal period, the HRCT findings were not evaluated according to the BPD severity of the cases; the pathologies detected are given in general percentages [24, 26, 28, 29]. In the literature, there were studies comparing HRCT values with clinical data. Ochiai et al. [8] found a correlation between the HRCT score, the clinical score, and the duration of oxygen therapy. In the other study performed by Aukland et al. [30], the HRCT score of cases with moderate and severe BPD was significantly higher than that of cases with non-BPD or mild BPD; in cases with severe BPD, there were determined areas of increased opacity and decreased attenuation. In addition, it was determined that HRCT scores decreased as the neonatal gestational age and birth weight increased; also, the HRCT score increased when the neonatal oxygen requirement increased and pulmonary function tests deteriorated (forced expiratory volume in the first second (FEV1), forced expiratory flow (FEF) 50 and FEF 25-75 decreased). In our cases, the scoring of Ochiai et al. [8] was used for the evaluation of HRCT. According to this method of scoring, HRCT findings were mainly evaluated in three categories consisting of hyperexpansion, emphysema, and fibrosis. The average HRCT score of the cases with moderate BPD was 7.20 ± 4.05 and that of cases with severe BPD was 7.40 ± 2.84; the difference between the HRCT scores of moderate and severe BPD groups was not significant (*p* = 0.620). Besides, no significant correlation was found between the HRCT total scores of moderate and severe BPD cases and the clinical findings and drug use of cases between 6 months and 1 year.

## 5. Conclusion

Those data suggest that the clinic may not always be parallel to radiology in BPD patients and that the clinical course of infants with severe radiological data may be better or worse than expected. A contemporary definition of BPD that correlates with respiratory morbidity in childhood is needed [31]. Also, a new lung ultrasound technique for predicting the respiratory outcome in patients with BPD can be used instead of HRCT [32].

### 5.1. Limitations

In this study we used data from other hospitals. As a result of this, changes in management of newborns may have affected the results. Also, this study was conducted with a single radiologist evaluation. Evaluation by a second radiologist could have increased the value of the study. From another point of view, limitations of current definitions of BPD affected the results. However, we believe more studies with larger number of patients and with different scoring systems are necessary in order to evaluate the role of HRCT in the clinical evaluation and long-term prognosis of BPD.

## Figures and Tables

**Table 1:** BPD and HRCT score table.

Category	Variable	0	1	2
Hyperexpansion	Hyperexpansion	None	Focal	Global
	Mosaic pattern of lung attenuation	None	Unclear	Obvious
	Intercostal bulging	None	Unclear	Obvious
Emphysema	Number of bullae or blebs	None	Single	Multiple
	Size of bullae or blebs	None	≤5 mm	>5 mm
Fibrosis	Triangular subpleural opacities	None	1-3 lobes	4-6 lobes
	Deformity and thickening of the bronchovascular structure	Mild	Moderate	Severe
	Consolidation	None	Unclear	Obvious
Subjective evaluation		Mild	Moderate	Severe

**Table 2:** General characteristics of the cases in the neonatal period.

Features	Cases (*n*: 28)
Gender (male/female)	13/15
Place of birth (AUFM/outer centers)	15/13
Gestational age^∗^	30 ± 2.9
Birth weight^∗∗^	1372 ± 784
Mechanical ventilation time^*β*^	22.9 ± 16
CPAP duration^*β*^	15.3 ± 4.2
Hood oxygen uptake period^*β*^	12.0 ± 4.2
Total oxygen support duration^*β*^	50 ± 16.7
Mechanical ventilation duration of moderate and severe BPD^*β*^	26.6 ± 17.3
CPAP duration of cases with moderate and severe BPD^*β*^	16.7 ± 3.5
Hood duration of cases with moderate and severe BPD^*β*^	11.7 ± 3.9
Total oxygen support duration of cases with moderate and severe BPD^*β*^	54.9 ± 17.4

^∗^Average ± SD (week), ^∗∗^average ± SD (gram), and ^*β*^day.

**Table 3:** HRCT score of cases with moderate and severe BPD.

Case	HS	MPS	İBS	NBS	BSS	TSOS	DTBSS	CS	SES	Total points
1	1	2	0	1	1	0	0	0	1	6
2	1	2	1	0	0	1	2	1	2	10
3	0	1	0	0	0	0	0	0	0	1
4	1	2	0	0	0	1	1	0	1	6
5	1	1	0	0	0	0	0	0	0	2
6	1	2	0	2	2	0	0	0	1	8
7	1	2	1	1	1	0	0	0	1	7
8	0	1	0	0	0	0	0	0	0	1
9	1	2	0	1	1	1	1	0	1	8
10	1	2	0	1	1	0	0	0	1	6
11	1	2	1	2	1	0	0	0	1	8
12	1	1	1	0	0	0	0	0	1	4
13	1	2	1	2	1	1	1	2	1	12
14	1	2	1	2	1	2	1	2	2	14
15	1	2	1	0	0	1	1	2	2	10
16	1	2	2	0	0	1	1	1	1	9
17	1	2	0	2	1	0	1	0	1	8
18	2	2	1	0	0	1	2	0	2	10
19	1	1	0	2	1	1	0	0	1	7
20	1	2	1	2	1	1	0	0	1	9

HS: hyperexpansion score; MPS: mosaic pattern score; IBS: intercostal bulge score; NBS: number of bulla score; BSS: bulla size score; TSOS: triangular subpleural opacity score; DTBSS: deformity and thickening of the bronchovascular structure score; CS: consolidation score; SES: subjective evaluation score.

**Table 4:** Comparison of the neonatal period characteristics and HRCT total score of cases with moderate and severe BPD (*n*: 20).

	Duration (days)	HRCT	*p* ^∗^	*r* ^∗∗^
Oxygen	54.90 ± 17.40	7.30 ± 3.4	0.408	-0.196
MV	26.60 ± 17.30	7.30 ± 3.4	0.836	-0.05
Steroid therapy				
Yes (*n*: 9)		7.45 ± 3.64	0.789	
No (*n*: 11)		7.11 ± 3.30		
Diuretic therapy				
Yes (*n*: 20)		7.30 ± 3.4	0.194	
Birth weight (g)	1478 ± 882	7.30 ± 3.4	0.439	0.183

^∗^ *p* < 0.05, ^∗∗^*r*: Spearman correlation coefficient. MV: mechanical ventilation.

## Data Availability

All data generated or analyzed during this study are included in this publıshed article.
